# Evaluation of PVC-Type Insulation Foam Material for Cryogenic Applications

**DOI:** 10.3390/polym15061401

**Published:** 2023-03-11

**Authors:** Dae-Hee Kim, Jeong-Hyeon Kim, Hee-Tae Kim, Jeong-Dae Kim, Cengizhan Uluduz, Minjung Kim, Seul-Kee Kim, Jae-Myung Lee

**Affiliations:** 1 Department of Naval Architecture and Ocean Engineering, Pusan National University, Busan 46241, Republic of Korea; 2 Hydrogen Ship Technology Center, Pusan National University, Busan 46241, Republic of Korea; 3 Diab Korea, A505 SKY-Biz Tower, 97 Centum Jungang-Ro, Haeundae-gu, Busan 48058, Republic of Korea

**Keywords:** cargo containment system (CCS), mechanical characteristics, thermal conductivity, structure support, polyvinyl chloride (PVC)-type foam, polyurethane foam (PUF), cryogenic

## Abstract

With the International Maritime Organization (IMO) reinforcing environmental regulations on the shipbuilding industry, the demand for fuels, such as liquefied natural gas (LNG) and liquefied petroleum gas (LPG), has soared. Therefore, the demand for a Liquefied Gas Carrier for such LNG and LPG also increases. Recently, CCS carrier volume has been increasing, and damage to the lower CCS panel has occurred. To withstand liquefied gas loads, the CCSs should be fabricated using a material with improved mechanical strength and thermal performance compared with the conventional material. This study proposes a polyvinyl chloride (PVC)-type foam as an alternative to commercial polyurethane foam (PUF). The former material functions as both insulation and a support structure primarily for the LNG-carrier CCS. To investigate the effectiveness of the PVC-type foam for a low-temperature liquefied gas storage system, various cryogenic tests, namely tensile, compressive, impact, and thermal conductivity, are conducted. The results illustrate that the PVC-type foam proves stronger than PUF in mechanical performance (compressive, impact) across all temperatures. In the tensile test, there are reductions in strength with PVC-type foam but it meets CCS requirements. Therefore, it can serve as insulation and improve the overall CCS mechanical strength against increased loads under cryogenic temperatures. Additionally, PVC-type foam can serve as an alternative to other materials in various cryogenic applications.

## 1. Introduction

Since the MARPOL convention of 1973/1978, the International Maritime Organization (IMO) has implemented emission standards on the major environmental pollutants originating from vessels, namely NOx and SOx [1]. Recently, countries have been reinforcing these regulations to reduce the oxide content of ship fuels under the supervision of the IMO [2]. The increasingly restrictive regulations have spurred the development of several methods to reduce pollution, e.g., exhaust gas recirculation and selective catalytic reduction systems. However, the equipment cost of these methods is quite high. Hence, alternative forms of conventional fuels, which meet the abovementioned regulations, are being considered [3].

Natural gas (NG) is one such alternative. Generally, it can be used as liquefied natural gas (LNG). When NG is converted to LNG, its volume is reduced by more than 600-times. It is a clear, odorless, non-toxic, and non-corrosive cryogenic liquid at atmospheric pressure. Liquefied petroleum gas (LPG) is also a clean, abundant, and ecofriendly fuel. It can be easily condensed, packaged, stored, and utilized, and, hence, large amounts of energy can be stored and transported compactly. Therefore, the demand for LPG and LNG has increased, leading to a surge in the demand for LNG and LPG carriers [4,5]. The transportation and storage of LNG and LPG are significant concerns. LNG is transported at a cryogenic temperature (−163 °C), and LPG is transported at a subzero temperature (−42 °C). Additionally, they are stored in the state that they are transported in. Therefore, LNG and LPG carriers ought to be equipped with specially designed cargo containment systems (CCSs) to maintain cryogenic or subzero temperatures.

**Figure 1:**
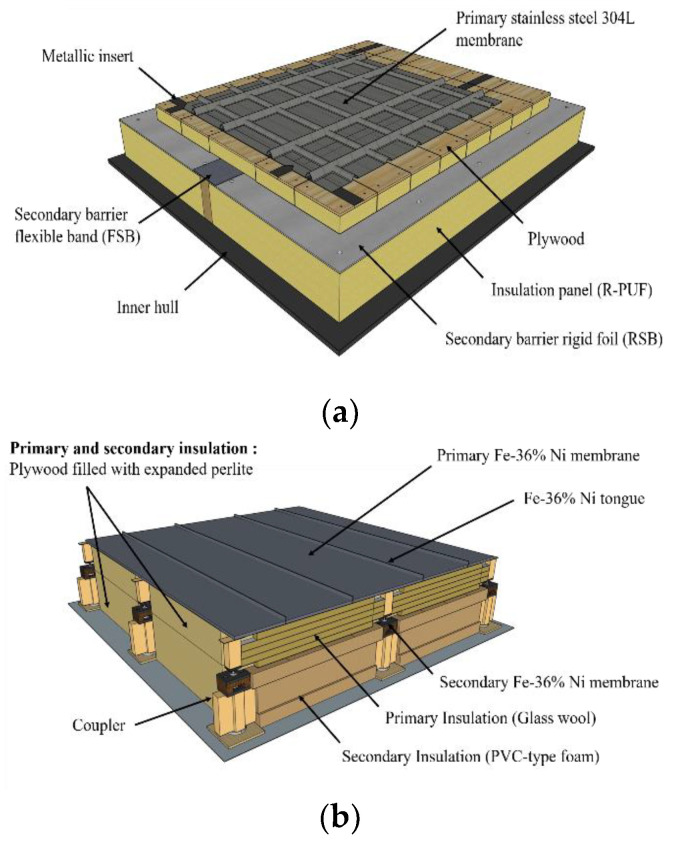
(**a**) Mark III and (**b**) NO96 liquefied natural gas (LNG) cargo containment system (CCS).

The LNG CCS is a membrane-type tank and is largely subdivided into two types: Mark III and NO96 [6]. As illustrated in Figure 1a, Mark III is composed of a primary stainless-steel 304 L membrane positioned on top of an insulation panel that incorporates a composite secondary barrier. The insulation consists of a reinforced polyurethane foam (R-PUF) [7]. In the NO96 system, both the primary and secondary barriers are made of thin sheets of invar, a Ni alloy. The shape of the box made of plywood filled with pearlite glass wool is shown in Figure 1b. As the NO96 panel boxes are contained in a sliding structure, the loads generated by the movement of the panels must be supported; this is achieved with the PVC-type foam located at the sidewall (Figure 1b). It simultaneously serves as an insulation and a liner to safely transport LNG at −163 °C [8].

**Figure 2:**
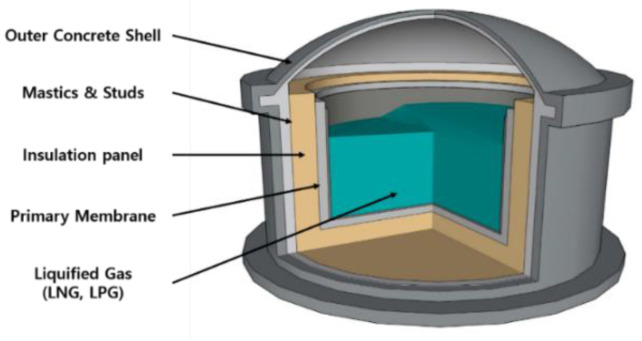
Approximate schematic diagram of a common storage tank (LNG and liquefied petroleum gas (LPG)).

The LPG CCS is similar to the LNG CCS; however, the latter is an independent tank type A defined by the IMO. The cargo tank consists of outer shell plates, bulkheads, stiffeners, web frames, and stringers. The outside of the tank is insulated in a layer of polyurethane foam (PUF). Therefore, the insulation panel is a critical part of both LNG and LPG CCSs [9]. The inner panels of the LNG and LPG tanks have a large temperature difference from the outer ones and are subject to heavy loads. Proper insulating materials must be used to maintain the inner temperature and load resistance. A schematic of the LNG and LPG tanks is depicted in Figure 2 [10,11].

As observed in the figure, all materials and structures in the primary membrane, insulation panel, etc., are the same as LNG CCS Mark III, except for R-PUF. PUFs are used as insulation for land storage tanks instead of R-PUF [11]. PUF and PVC foams are used as insulation materials in the CCS and storage tank. Currently, PUF is preferred as the main structural material in various cryogenic devices and has several applications—as insulation in cryogenic storage tanks for ship and rocket propulsion systems, core material in construction panels, and thermal insulation of refrigerated vehicles. Recently, South Korea has been developing an LNG CCS line called KC series, with PUF as an insulation material. PUF has already been employed for insulation in KC-1 [12].

**Figure 3:**
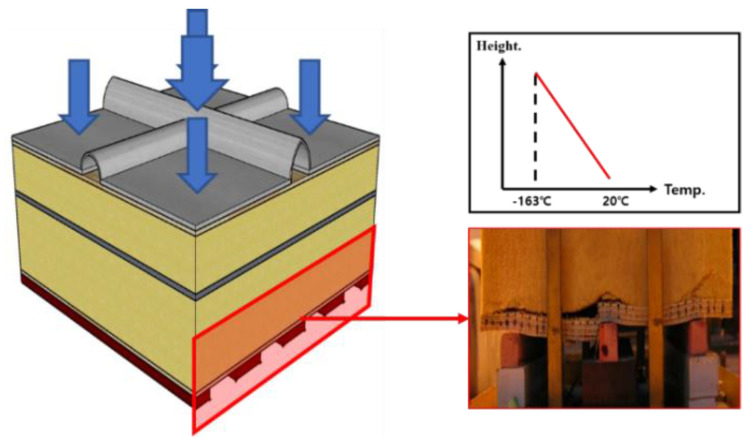
Stress concentration at the bottom of an insulation panel and the corresponding temperature gradient.

As mentioned above, PUF finds applications in several fields. In particular, it is widely used in CCS. However, among them, engineers have observed stress concentration at the bottom of the insulation of the Mark III CCS. The insulation panels were attached to the inner walls of the containment using a series of mastic ropes (Figure 3) [13,14]. This phenomenon occurred because of a temperature gradient along the depth or height of the CCS as well as gaps between the mastic ropes (Figure 3) [15,16]. This phenomenon could cause severe damage or collapse of the structure. Therefore, in the lower part of the insulation panel, indicated by the red section in Figure 3, the mechanical strength of the material plays a more important role than the thermal performance. Larger carrier vessels and other larger ships are being commissioned to efficiently transport more consignments. With the increase in demand for LNG and LPG or heavier freight, the acting loads on the CCS also increase. Hence, the components of a CCS or storage tank require greater mechanical strength and thermal performance [17]. Therefore, evaluation of the strength of the insulation panel structures is important [18].

Accordingly, PVC-type foam is used to improve the mechanical strength of NO96-type LNG CCS as it is a high-strength, low-weight polymeric material, with closed cell structures. Hence, it has been widely used as an insulation and reinforcing material for LNG CCS.

In the past few decades, several studies have been conducted to evaluate the mechanical strength of polymer insulation materials under cryogenic environments. Park et al. (2016) investigated the effect of the blowing agent on the cryogenic temperature mechanical properties of glass-fiber-reinforced polyurethane foams. The test was conducted at various cryogenic temperatures and strain rates. The compressive elastic modulus and compressive strength increased as the temperature decreased. At −163 °C, the fluctuations became more severe than those at other temperatures. In the aspect of blowing agent, the compressive stress of the CO2-blown reinforced polymer foam was higher than that of the HFC-245fa-blown reinforced polyurethane foam [19]. Son et al. (2019) investigated the synthesis of polyurethane foam for application to cryogenic environments. Polyurethane foams were manufactured, and samples were subjected to mechanical tests to investigate the mechanical properties of the polyurethane foam within a temperature range of 25 °C to −163 °C. In addition, thermal and microstructural investigations were conducted. The compression behavior of polyurethane foam was also analyzed. Regardless of the blowing agent content, all the polyurethane foams showed better compression strength at cryogenic temperatures than at room temperature. The compression strength was improved from at least 76% to 199% [20]. Marsavina and Linul (2022) investigated the fracture toughness of rigid polymeric foams. The different approaches for estimating the fracture toughness of polymeric foams were reviewed. Focus was given on the parameters influencing the fracture toughness of polymeric foams, such as specimen type, solid material, density, loading speed, size effect, and temperature [21]. Including representative research activities, several research outcomes have been reported on the effect of cryogenic temperatures on the mechanical characteristics of polymeric foams [21]. However, most of the research has been focused on polyurethane foam, which is already applied in cryogenic liquid gas storage systems [22,23,24,25,26,27,28]. Few studies have reported the mechanical properties and performance of PVC-type foam under cryogenic environments [8].

The primary objective of this study is to closely examine the material properties and insulation performance of the PVC-type foam under cryogenic temperatures by conducting tensile, compressive, and impact tests. In addition, thermal conductivity, which is an important material property of the foam insulation, was investigated. Finally, the criteria for the further applications of the PVC-type foam in various fields are presented.

## 2. Materials and Methods

### 2.1. Test Specimen and Apparatus

To investigate the mechanical properties of the PVC-type foam, thermal conductivity, compressive, tensile, and impact tests were conducted under ambient and cryogenic temperatures. The PVC-type foam was commercially manufactured (Diab, Helsingborg, Sweden). The tested PVC foam material was manufactured using PVC, isocyanates, anhydrides, and blowing agents. The PVC resin is a thermoplastic component, and isocyanates contribute to the fabrication of a new thermoplastic polymer as well as a thermoset to improve mechanical properties. In addition, anhydride is a plasticizer and contributor to the gas phase. The PVC foam is manufactured in various steps. The raw materials are mixed to a homogeneous plastisol. Then, the plastisol is filled into molds, which are then put into presses and subjected to heat and pressure. This creates a rubbery, small, and visually solid block. Then, it is expanded and further cured in heated water and a steam environment in different chambers with varying temperatures. In addition, polyurethane foam was manufactured directly by synthesizing basic raw materials, such as polyol, isocyanate, and blowing agents. The polyol and isocyanate were mixed at a fixed ratio of 700:800. The polyol, isocyanate, and blowing agent were mixed by a homogenizer at 4000 rpm for approximately 60 s to synthesize a homogenous PUF solution. Then, the solution was poured into an open mold (length × width × height = 300 mm × 300 mm × 250 mm). It was then foamed and cured at ambient temperature (20 °C). Finally, after 24 h, the solid PUF was machined to a different size for each test [29,30]. The dimensions of the thermal conductivity test specimen were 280 mm × 280 mm × 25 mm, while those of the compressive and impact test specimen were 50 mm × 50 mm × 25 mm, according to KS M ISO 844 and KS M ISO 6603. The tensile test specimen was also manufactured according to KS M ISO 1926. Table 1 shows the average density of the PUF and PVC-type foam for each test. A universal testing machine (UTM) with a cryogenic chamber was used for the compressive and tensile tests. The reason for using the cryogenic chamber was to maintain the cryogenic conditions with liquid nitrogen (LN2) and an automatic temperature control box. The thermal conductivity was measured using a heat flow meter (HFM436, NETZSCH).

#### 2.1.1. Thermal Conductivity

The thermal conductivity was measured using a heat flow meter HFM436 (NETZSCH). This equipment consisted of upper and lower plates; the upper plate had a higher temperature than the lower plate, and the specimen was placed between the plates. The dimensions of the specimen were depicted before. Subsequently, heat flow was generated to measure the thermal conductivity according to KS L 9016.

#### 2.1.2. Tensile Test

In the tensile test, the specimens were precooled according to the test temperature. Subsequently, they were positioned in the UTM equipped with a tensile jig. After attaching the strain gauge, the tensile test was performed at a tensile test speed of (5 ± 1) mm/min according to the KS M ISO 1926.

#### 2.1.3. Compressive Test

In the compressive test, after the specimens were precooled, similar to the tensile test, they were positioned in the UTM equipped with a compressive jig. Subsequently, the test speed was set to 2.5 mm/min, which was approximately 10% of the thickness of the specimen according to KS M ISO 844, and the experiment was conducted.

#### 2.1.4. Impact Test

The impact test was conducted on Instron CEAST9340. The test was conducted using two different impact energies fitted to two specimens with different densities under a varying temperature, ranging from room to cryogenic temperatures. The criterion for selecting the impact energy used energy absorption as a measure for estimating the impact performance. To calculate the absorption energy, the force before the densification region was required [31]. Therefore, the two impact energies near the densification region were selected in this study and determined to be 30 and 60 J.

Subsequently, a square impactor, which had a cross-section similar to that of the specimen, was used. The weight mounted on the impact equipment was approximately 30 kg. The low-temperature impact test specimens were precooled for approximately 1 h before the impact experiment, similar to the tensile and compressive tests. The impact experiments were conducted within 5 s of removing the specimens from the cooled area, in accordance with KS M ISO 6603.

#### 2.1.5. Scanning Electron Microscopy

Foam density is a very important parameter because it alters the number and size of the cell. Hence, the present study controlled the foam density by controlling the chemical reaction between blowing agent and isocyanate [32,33,34]. The cell morphology of the PUFs and PVC-type foams was observed using scanning electron microscopy (SEM; SUPRA25 (ZEISS, Jena, Germany) coupled with an energy-dispersive X-ray spectrometer). The SEM consisted of an electron gun, lens, chamber, and detector. The electron gun generated an electron beam and focused it on the inspection object at 10 kV. In addition, the lens controlled the size and intensity of the beam. Testing samples for SEM analysis were cut to 10 mm × 10 mm × 5 mm by a sharp razor blade to minimize damage to the cell structures [35,36]. The prepared testing samples were coated for protection against heated electron beam.

### 2.2. Experiment Temperature Environment

This study calculates the insulation performance and mechanical properties of the PVC-type foam as compared to PUF by varying the temperature and density. The temperature was selected based on the liquefaction temperature of LNG (−163 °C) and LPG (−42 °C). Therefore, the test temperatures were 20, −50, −110, and −170 °C. Particularly, in a cryogenic experiment, to calculate the exact mechanical properties, the internal temperatures of the test specimen should be equal to the external temperature. Therefore, specimens were precooled to each temperature prior to testing.

## 3. Results and Discussion

### 3.1. Cellular Morphology of PUF and PVC-Type Foam

**Figure 4:**
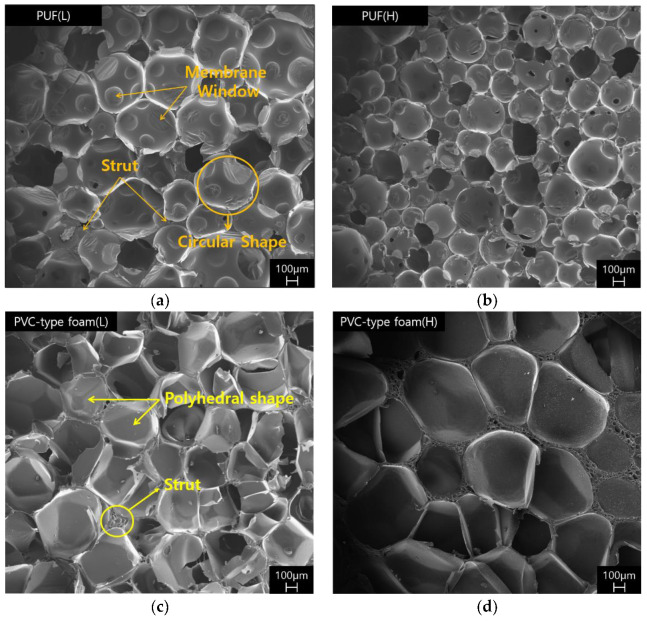
SEM images of microstructures: (**a**,**b**) PUF (L, H) and (**c**,**d**) PVC-type foam (L, H).

The changes in mechanical properties mentioned above are a result of the changes in the cell structure. Hence, the cell morphology had to be verified. Figure 4 displays the SEM images of the PUF (L, H) and PVC-type foam (L, H) at approximately 32× magnification. Photographs of the cell sizes and shapes of the specimens are presented in Figure 4.

The cell size was different for specimens with different densities. It should be, and sometimes is, an important parameter. However, most mechanical and thermal properties have a weak dependence on the cell size, and the cell shape is more important [34]. On the one hand, the PVC-type foam and the PUF specimens have a closed cell shape, according to previous studies [37], and the cell shape of PVC-type foam is polyhedral in topology, like the typical PVC foam. On the other hand, the cell shape of the PUF was approximately spherical and bears the same appearance as the typical polyurethane foam [37,38,39,40,41]

Table 2 summarizes the cell diameters of PVC-type foams and PUFs. Previous studies confirmed that if the PUF (rigid PUF) had larger diameters, it also had higher thermal conductivity [42,43]. PUF (L) had a larger diameter than PUF (H), which is consistent with previous studies. However, PVC-type foam exhibited the opposite result to the PUF. PVC-type foam (L) had a smaller diameter than PVC-type foam (H). At low densities, the PUF (L) and PVC-type foam (L) had nearly identical diameters. However, at high densities, PVC-type foam (H) had approximately 120% larger diameter than neat PUFs. Hence, PVC-type foam and PUF have different cell size tendencies.

**Figure 5:**
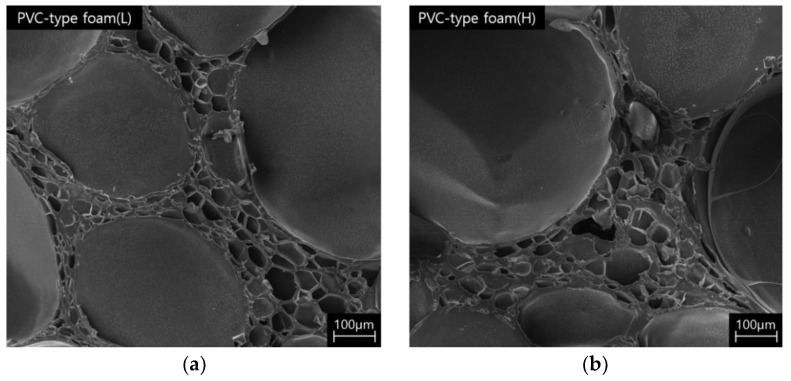
Micrograph of a PVC-type foam with a wide distribution of cell sizes: (**a**) PVC-type foam (L) and (**b**) PVC-type foam (H).

**Figure 6:**
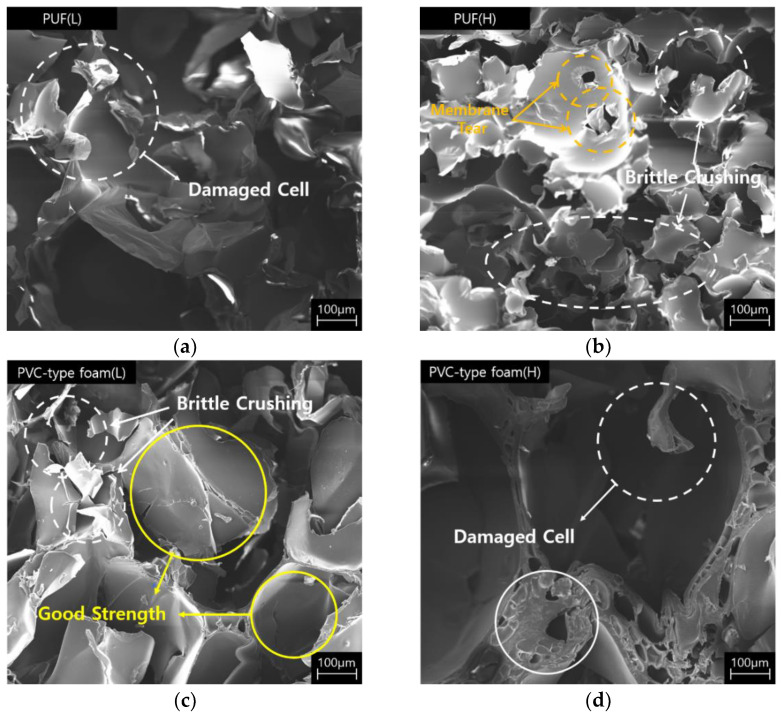
Micrographs of the specimens after the cryogenic compressive test (−170 °C): (**a**) PUF (L), (**b**) PUF (H), (**c**) PVC-type foam (L), and (**d**) PVC-type foam (H).

Figure 5 presents the SEM images of PVC-type foam observed at approximately 82× magnification. As presented in Figure 5, small cells of different sizes are generated within a small space between the formed cells. These shapes can be observed when the cells are random and of different sizes. Cells coarsen if the cells become connected (pore wall between them breaks) or if gas is generated in the cell. Subsequently, the many-sided cells grow, while the few-sided ones shrink, and this consequently results in an increasingly inhomogeneous structure. In this process, the diffusion rate of the cells is affected by pressure [34]. Therefore, unlike the PUF that was foamed and cured at ambient temperatures, PVC-type foam was processed at high pressures and temperatures. Therefore, while the cells were foaming, the cell diffusion rate was affected by the processing heat and pressure. Hence, this morphology appeared in PVC-type foam. These shapes can also be observed in Figure 6 at approximately 82× magnification. A few cell walls or cells in the PUF and PVC-type foam were damaged. The yellow circles in Figure 6 are the parts of the sparse distribution of cell sizes from Figure 5 that were confirmed to not have suffered severe damage and that retained the original shape. Therefore, the fact that these parts affected the compressive and impact strengths under a varying temperature (from room to cryogenic) is verified.

As mentioned in Section 2, the production of the foam-type material involves a blowing process. The thermal and mechanical properties may vary with the amounts of the true material and blowing agent. This is called the true density, which allows one to know the amount of the actual material. The results of the true density calculations of this PVC-type foam and PUF are listed in Table 3.

At low densities, the PVC-type foam had a larger true volume occupied by the material relative to the similar mass than that of the PUF. Hence, its true density is lower than that of neat PUF. In the case of a high density, both exhibit similar values. As mentioned above, as the SEM images and cell size had different characteristics according to the densities of the two materials, the true density will also differ. Moreover, we considered that the true density affects the strength of the cryogenic environment for materials with high densities.

### 3.2. Thermal Conductivity

**Figure 7:**
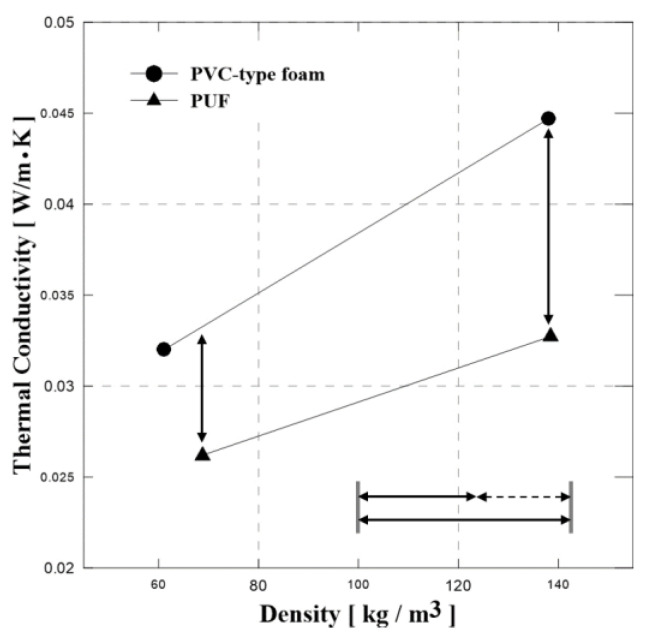
Comparison of thermal conductivity between PVC-type foam and PUF.

In general, thermal conductivity determines the insulating performance of the material. Materials with a high thermal conductivity are used as heat dissipaters, whereas materials with a low thermal conductivity are used as insulation. Therefore, we measured the thermal conductivity of the PVC-type foam and PUF using the HFM. In general, density is one of the most important parameters for determining the mechanical and thermal properties of foam materials. The low density of foam materials results in low thermal conductivity, thereby providing better insulating performance. To compare the thermal conductivity of PUF and PVC-type foam, foam materials with similar densities were selected. Figure 7 shows a comparison of thermal conductivity between PVC foam and neat PUF. In addition, Table 4 presents the measured thermal conductivity of PVC-type foam and neat PUF. Results showed that the thermal conductivity of neat PUF was lower than that of PVC-type foam. As shown in Figure 7, the difference in thermal conductivity between PUF and PVC-type foam is much more significant with increasing density. At low densities, the thermal conductivity of PUF was approximately 20% lower than that of PVC-type foam. In addition, the thermal conductivity of neat PUF was approximately 35% lower than that of PVC-type foam at relatively high densities.

### 3.3. Tensile Test

**Figure 8:**
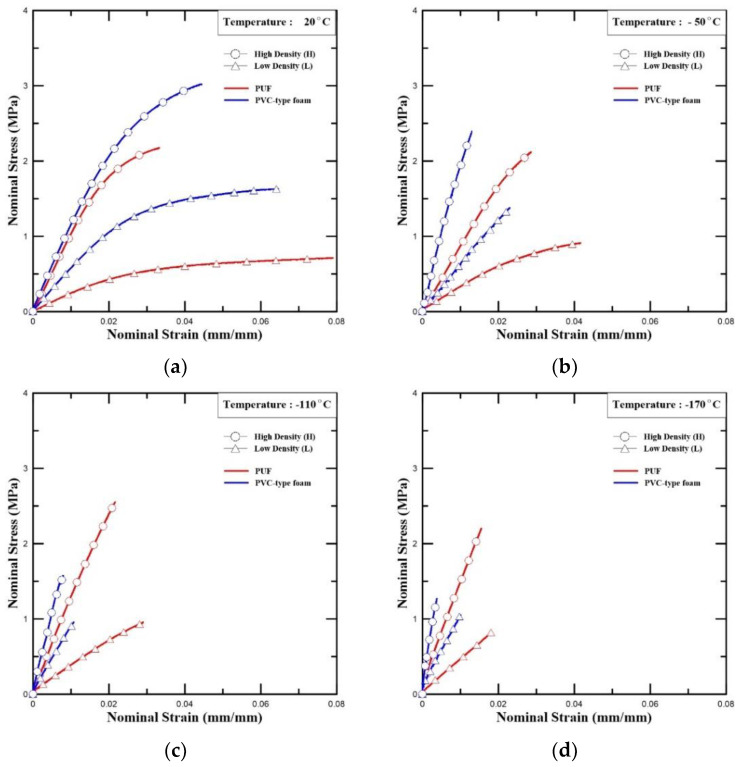
Stress–strain curve of the PUF (H, L) and PVC-type foam (H, L) with different temperatures: (**a**) 20 °C, (**b**) −50 °C, (**c**) −110 °C, and (**d**) −170 °C (tensile test).

Figure 8 shows the results of the test. Generally, polymer foam materials with higher densities possess higher strength than those with lower densities [44]. Therefore, as illustrated in Figure 8, the tensile strengths of both PVC-type foam and PUF, which had higher densities, were better than those with lower densities.

In Figure 8, at room temperature and −50 °C, the tensile strength of PVC-type foam was confirmed to be comparatively higher than that of PUF. However, in contrast, the tensile strength of PUF (H) was higher than that of PVC-type foam (H) at low temperatures (−110 °C and −170 °C).

**Figure 9:**
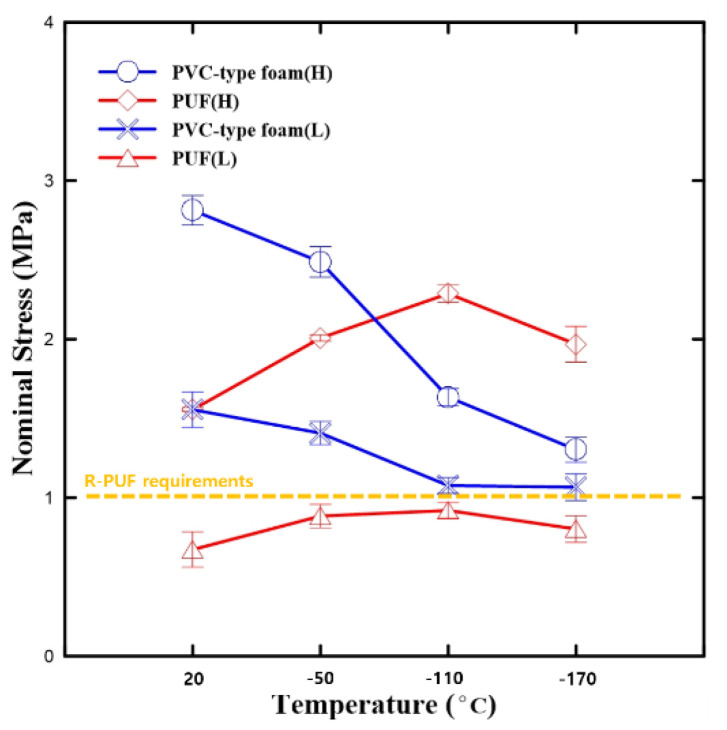
Nominal stress vs. temperature diagram comparing the tensile strengths of PVC-type foam and the PUF specimens with respect to R-PUF requirements at low and high temperatures.

Figure 9 illustrates the trend of the PVC-type foam, indicated by blue lines, which decrease steadily. The curve decreased between −50 and −110 °C. The red lines of the PUF increase until −110 °C and then decrease. The yellow grid line in Figure 9 represents the tensile strength requirement of the insulation material when using R-PUF insulation in the CCS [7]. Except for low-density PUF, PVC-type foam (H, L) and PUF (H) were located higher than the yellow grid line, indicating the requirements for CCS. Therefore, although the strength of the PVC-type foam decreased as the temperature decreased, it satisfied the standard strength criterion.

Table 5 lists the tensile elastic modulus and yield stress of the specimens. The values in the table are based on the averaged test results. In the case of PUFs, the yield stress increased to −110 °C and had the highest yield stress at that temperature. In contrast, the yield stress of PVC-type foam tended to decrease as the temperature decreased.

As shown in Figure 8 and Table 5, the elastic modulus increases as the temperature decreases. The tensile elastic modulus increased up to 130% and 220% for the low densities and high densities, respectively, of the PVC-type foam at cryogenic temperatures. In the strength aspect, the lower the temperature, the weaker the PVC-type foams. Although it had lower strength at lower temperatures, its strength was higher than the neat PUF’s highest strength at low density [45].

### 3.4. Compressive Test

**Figure 10:**
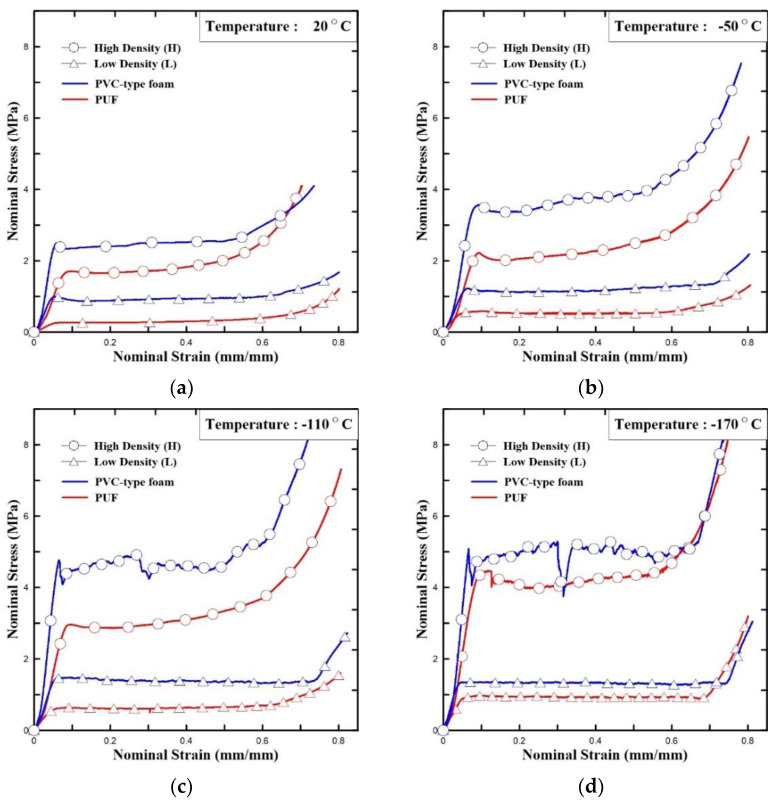
Stress–strain curve of the PUF(H, L) and PVC-type foam(H, L) at different temperatures: (**a**) 20 °C, (**b**) −50 °C, (**c**) −110 °C, and (**d**) −170 °C (compressive test).

Table 6 contains photographs of specimens after the compressive test at room and cryogenic temperatures, where the surfaces of specimens with the most marked differences are indicated. The specimens of both PUF and PVC-type foam were evenly deformed without any damage/failure at room to low temperatures (−50 to −110 °C). However, the breakage can be clearly observed at −170 °C in the cryogenic range. The PUF and PVC-type foam specimens were crushed and fractured. However, the neat PUF specimen was more severely crushed and fractured than the PVC-type foam. This phenomenon is due to the embrittlement of cellular structures at cryogenic temperatures [6,7,8]. Therefore, it can be confirmed that there is a critical point of cellular structure embrittlement, which is approximately −170°C. Compared with these results, the cellular structure is not severely embrittled in the PVC-type foam. Subsequently, a strain–stress curve with experimental data is plotted in Figure 10. In Figure 10, the left-hand-side axis is the nominal stress, which is expressed as follows:

(1) $$\sigma =\frac{P}{A_{0}}$$

where *P* is the load applied to the specimen and *A*_0_ is the initial section area. The nominal compressive strain is expressed as follows:

(2) $$\varepsilon =\frac{h_{0}-h\;}{h_{0}}$$

where *h* is the height of the specimen after undergoing a strain *ε* and *h*_0_ is the original height. We observed three characteristic phases: a linear elastic region, plateau region, and region of sharp increase. The linear elastic region is used for calculating the elastic modulus. A small decrease called strain softening occurred before the plateau region. The stress strongly fluctuated and unexpectedly decreased—a phenomenon known as brittle crushing. These characteristics became more prominent as the experimental temperature was reduced owing to the embrittlement of the cell wall. The sharp rise occurred due to densification because the cells of the material collapsed and became compressed [41,45,46].

The four foam specimens exhibited better compressive performance at low temperatures than at room temperature, which is in line with the results of previous studies [46]. Therefore, in Figure 10, the compressive strengths and elastic modulus of PUF and PVC-type foam increase as the temperature decreases. Subsequently, as observed in Figure 10, PVC-type foam had a higher compressive strength than neat PUF. Further, the gap between PVC-type foam and PUF with a similar density tended to narrow as the temperature decreased. The linear slope at the beginning of the graph is called the elastic modulus, and the elastic modulus of the PVC-type foam is generally larger than the PUF at all temperatures. The average compressive strength was also greater than that of the PUF having the same density. These results are confirmed not only by Figure 10 but also by Table 7, highlighting the average values of the test results. Compressive yield stresses of the PUF were enhanced by approximately 150% at cryogenic temperatures than at room temperature. Likewise, the compressive elastic modulus also increased by approximately 100%. Yield stresses of the PVC-type foam increased by approximately 70% at cryogenic temperatures for low densities. Similarly, the compressive elastic modulus also increased by approximately 100%.

**Figure 11:**
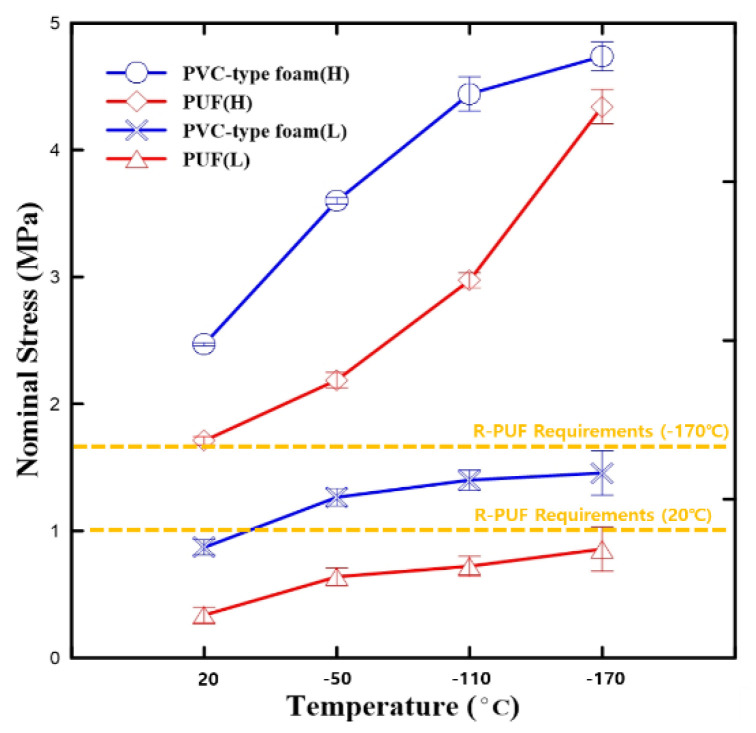
Nominal stress vs. temperature diagram comparing the compressive strengths of PVC-type foam and the PUF specimens with respect to R-PUF requirements at low and high temperatures.

Two standard compressive strength criteria exist for the optimal CCS using R-PUF at 20 °C and –170 °C [7]. The standard for –170 °C is higher than that for 20 °C as the embrittlement occurred. As indicated by the results in Figure 11, neither neat PUF nor PVC-type foam meet the criteria at low densities. However, as the generally used insulation density for the CCS is more similar to high density, the compressive stress of both neat PUF and PVC-type foam at high density satisfied both the two standard criteria for all temperatures.

### 3.5. Impact Behavior

**Figure 12:**
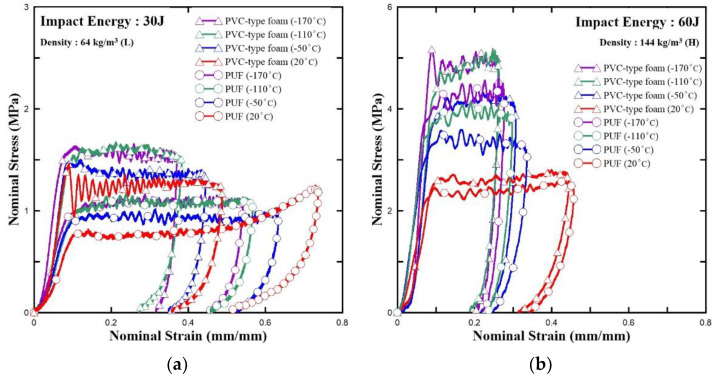
Stress–strain curve based on the results of the impact test: (**a**) 30 J and (**b**) 60 J of impact energy.

Larger cargo ships have greater freight capacity. Therefore, the contents of the tank have a significant impact on not only the vessel’s tank but also on the large storage tanks on land. This necessitates impact performance data, and, hence, the impact test was conducted. The results are presented in Figure 12.

**Figure 13:**
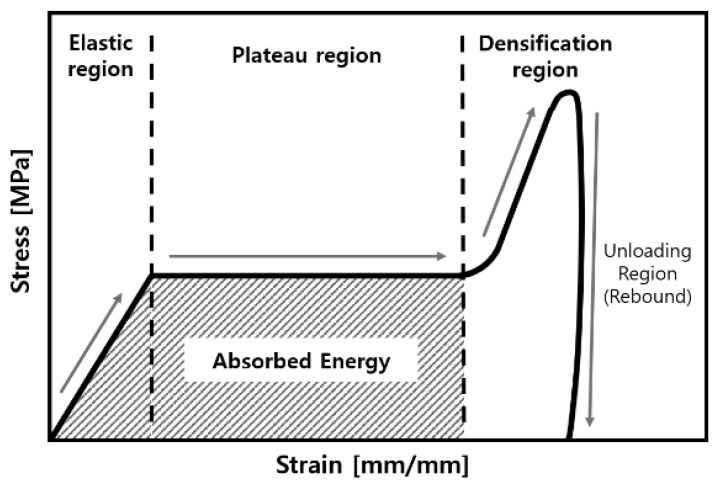
Stress–strain curve based on the results of the impact test.

As presented in Figure 12 and Figure 13 have four regions: linear, plateau, densification, and unloading. This figure shows the typical impact behavior of a foam-type material [47]. Similar to the abovementioned elastic region, it maintained linearity because the walls of the cells were bent and stretched [48]. Fluctuations occurred in the plateau region as the cells were damaged and crumbled by buckling. Subsequently, the cell walls and cell struts crumpled together. This caused the creation of the densification region. Next, the appearance of this region confirmed that stress rapidly decreased in the unloading region as the initial dynamic energy was removed [29]. Notably, this is similar to the compressive behavior. In Figure 12, the PVC-type foam is stronger than the PUF at both densities. Moreover, if the PUF and PVC-type foam have high density, they have higher strength. However, as the temperature increases, the strength decreases since the material becomes more brittle with a decrease in temperature. Therefore, the linear modulus of elasticity and yield stress increased.

Figure 13 illustrates the force–displacement curve of the PUFs and PVC-type foams with different densities at various temperatures and impact energies. Between the elastic region and the early segment of the plateau region, a point exists where the curve gradually linearly increases and then sharply decreases. This point is called the initial peak point, and the corresponding force is called the initial peak force. This force also increases as the density increases and the test temperature decreases [31].

Table 8 lists the average values of the test results. The higher the initial peak force, the higher the yield stresses and impact elastic modulus. Similar to previous studies, the findings of our study confirm that the PUFs and PVC-type foams both have a higher initial peak force if the temperature is reduced and the density is increased. Subsequently, PVC-type foams had a higher initial peak force than the PUFs under all the test conditions (Figure 12 and Table 8).

**Figure 14:**
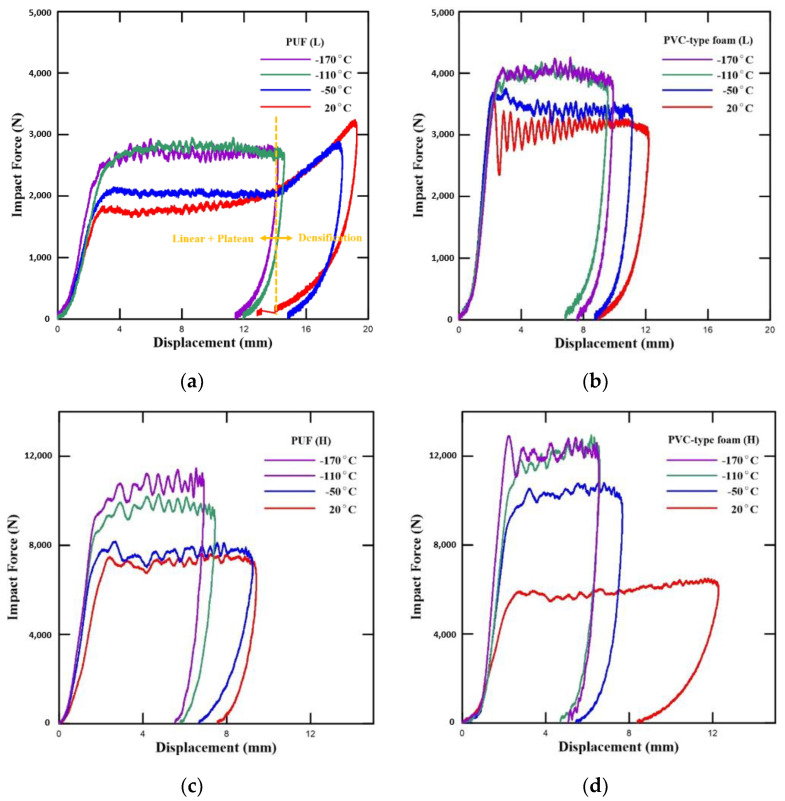
Force–displacement curves at 30 J (**a**,**b**) and 60 J (**c**,**d**) based on temperature: (**a**) neat PUF (L), (**b**) PVC-type foam (L), (**c**) Neat PUF (H), (**d**) PVC-type foam (H).

PVC-type foam also exhibited similar values with the PUF with high densities at room temperature. However, the values of PVC-type foam were evidently higher than that of the PUF by approximately 30% at −170 °C, while PVC-type foam in low density had a higher initial peak force up to approximately 88% at room temperature and up to approximately 48% at cryogenic temperature −170 °C. Although the differences were decreased by decreasing the test temperature, peak force of the PVC-type foams was higher than that of the PUF, as shown in Figure 14.

**Figure 15:**
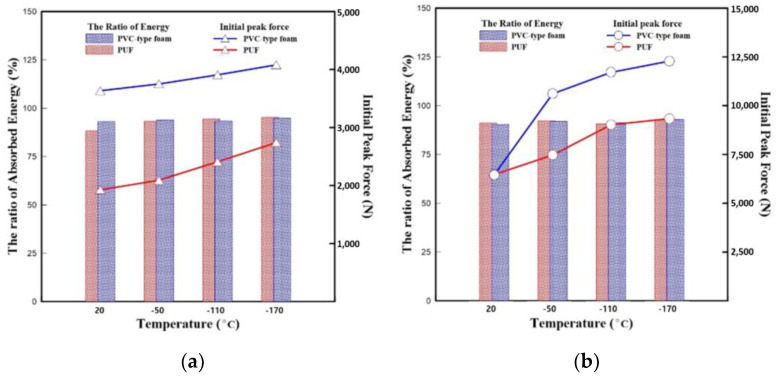
Comparison of specimens in terms of the initial peak force and the ratio of the absorbed energy by temperature: (**a**) for low densities (L), (**b**) for high densities (H).

Table 8 and the left-hand side of Figure 15 present the absorption energy. The energy absorbed per unit volume due to a strain ε is calculated by the area under the stress–strain curve. It can be expressed as follows:

(3) $$E_{absorbed}=\int_{0}^{\varepsilon }\sigma \left(\varepsilon \right)\;d\varepsilon ,$$

where *σ*(*ε*) is the stress as a function of strain, and the strain range is 0—*ε*, which is immediately before the start of the densification. When tested with the initial set impact energy, if the specimens are completely destroyed in the plateau region, the absorption energy can be obtained by integrating the stress value in the strain from the beginning to the end. This energy represents the area of the closed section of the graph. The obtained absorption energy is expressed as a ratio of the total impact energy. As the test temperature decreases, the ratio increases. This tendency was observed in previous studies as well, which tested composite or polymeric materials in cryogenic environments [48].

## 4. Conclusions

This study investigated the mechanical and thermal characteristics of neat PUF and PVC-type foam, which offer different densities from room to cryogenic temperatures. The results obtained from the present study are summarized as follows.

- The thermal conductivity test indicates that PUF offers better insulation performance and presents a thermal conductivity approximately 35% lower at a density of 144 kg/m^3^ and 20% lower at a density of approximately 64 kg/m^3^ than PVC-type foam.
- The results of a compressive test on PVC-type foam with a high density (144 kg/m^3^) exhibited that brittle crushing causes a significant decrease in target material strength. Additionally, the neat PUF tends to splinter into several pieces, whereas PVC-type foam develops dents. Thus, the PVC-type foams possess elevated strength over the PUFs.
- In the tensile test, the tensile strength and elastic modulus increase as temperature decreases. In both materials, a density of approximately 140 kg/m^3^ allows for a higher strength than at a density of approximately 64 kg/m^3^. High-density PVC-type foam is approximately 30% weaker than PUF at –110 °C or lower temperatures, whereas PVC-type foam is approximately 40% stronger than PUF at all temperatures.
- Overall, the impact test conveys that the initial peak forces of PVC-type foam are higher than those of PUF, while the absorption energy ratios are similar. Hence, PVC-type foam offers tangible advantages over PUFs in terms of impact resistance.

This study’s results present an opportunity for using PVC-type foam, which satisfied a high-strength requirement, even at low temperature. Additionally, PVC-type foam offers substantial strength with low density. This serves the trend of increasing CCS wall thickness. Although PVC-type foam thermal conductivity was higher than that of PUF, all mechanical strength aspects (tensile, compressive, and impact) were stronger in the former. Consequently, given that PVC-type foam provides higher strength, it represents a more advantageous alternative insulation material/panel (for cryogenic temperatures). Furthermore, it can fulfill not only CCS needs but also other applications that require low density, high strength, and cryogenic environments.

## Figures and Tables

**Table 1:** Average density of the PUF and PVC-type foam for each test.

Test Method	Test Material	Density (kg/mm^3^)	Specimen Thickness (mm)
Insulation	PUF	68.8 (L)	25
		138.4 (H)	
	PVC-type foam	61.1 (L)	
		138.1 (H)	
Compressive & Impact Test	PUF	64 (L)	25
		144 (H)	
	PVC-type foam	64 (L)	
		144 (H)	
Tensile test	PUF	64 (L)	15
		144 (H)	
	PVC-type foam	64 (L)	
		144 (H)	

**Table 2:** Cell diameter sizes of neat PUFs and PVC-type foams.

Material Type	Density (kg/m^3^)	Avg. Diameter (µm)	Standard Deviation	Max. Diameter (µm)
PUF	144 (H)	227.660	55.182	306.667
	64 (L)	388.138	92.211	581.513
PVC-type foam	144(H)	509.957	118.880	522.595
	64 (L)	383.646	67.543	306.667

**Table 3:** Results of the true densities of the two types of materials.

Material Type	Average True Volume (cm^3^)	Standard Deviation	Average True Density (kg/m^3^)	Standard Deviation
PUF (H)	46.6257	0.2158	0.1480	0.0007
PVC-type foam (H)	46.3865	0.0059	0.1492	0.0000
PUF (L)	50.1830	0.1492	0.0729	0.0002
PVC-type foam (L)	54.1881	0.0245	0.0650	0.0000

**Table 4:** Thermal conductivity of PVC-type foam and neat PUF.

Material	Thermal Conductivity (Average)	Average Density (kg/mm^3^)
PUF (H)	0.0327	138.4
PVC-type foam (H)	0.0447	138.1
PUF (L)	0.0262	68.8
PVC-type foam (L)	0.0320	62.1

**Table 5:** Test results of tensile Young’s modulus and yield stress.

Material	Temperature	Young’s Modulus (MPa)	Standard Deviation	Yield Stress (MPa)	Standard Deviation
PUF (L)	20 °C	19.347	1.883	0.672	0.074
	−50 °C	25.759	2.073	0.884	0.049
	−110 °C	33.376	2.671	0.919	0.033
	−170 °C	44.178	1.138	0.802	0.056
PUF (H)	20 °C	67.361	2.847	1.555	0.065
	−50 °C	89.619	4.563	2.007	0.097
	−110 °C	104.698	37.951	2.287	0.266
	−170 °C	142.142	17.354	1.968	0.563
PVC-type foam (L)	20 °C	50.939	0.814	1.555	0.065
	−50 °C	60.154	5.899	1.407	0.054
	−110 °C	79.404	5.880	1.076	0.186
	−170 °C	116.492	14.709	1.066	0.099
PVC-type foam (H)	20 °C	95.162	5.915	2.813	0.232
	−50 °C	170.611	12.569	2.487	0.241
	−110 °C	183.312	21.274	1.634	0.135
	−170 °C	306.282	16.078	1.302	0.198

**Table 6:** Photographs of specimens after the compressive test.

Temperature	Material	
20 °C	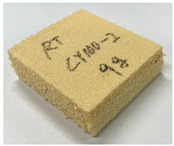 PVC-type foam (H)	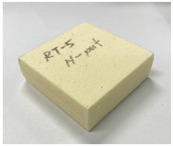 PUF (H)
	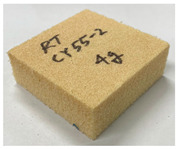 PVC-type foam (L)	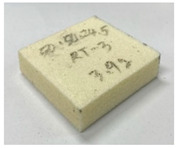 PUF (L)
−170 °C	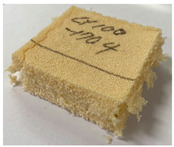 PVC-type foam (H)	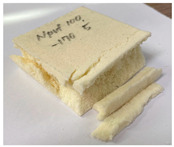 PUF (H)
	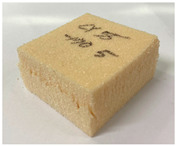 PVC-type foam (L)	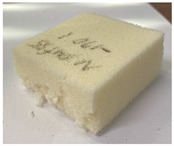 PUF (L)

**Table 7:** Test results of compressive Young’s modulus and yield stress.

Material	Temperature	Young’s Modulus (MPa)	Standard Deviation	Yield Stress (MPa)	Standard Deviation
PUF (L)	20 °C	6.537	2.729	0.337	0.060
	−50 °C	9.097	0.839	0.639	0.070
	−110 °C	9.857	0.079	0.722	0.079
	−170 °C	13.626	3.444	0.857	0.175
PUF (H)	20 °C	21.767	4.012	1.710	0.065
	−50 °C	23.644	2.103	2.188	0.121
	−110 °C	39.481	4.742	2.973	0.119
	−170 °C	43.557	2.524	4.342	0.271
PVC-type foam (L)	20 °C	17.259	4.306	0.870	0.284
	−50 °C	22.433	1.640	1.265	0.033
	−110 °C	24.947	2.106	1.400	0.051
	−170 °C	34.523	4.031	1.456	0.067
PVC-type foam (H)	20 °C	41.474	4.148	2.469	0.029
	−50 °C	55.169	6.119	3.600	0.063
	−110 °C	64.573	10.152	4.442	0.337
	−170 °C	73.322	2.083	4.738	0.285

**Table 8:** Test results of initial peak force and ratio of absorbed energy.

Material	Temperature	Initial Peak Force (N)	Standard Deviation	Ratio of Absorbed Energy (%)	Standard Deviation
PUF (L)	20 °C	1924.701	126.129	88.218	0.130
	−50 °C	2091.565	77.305	93.306	0.434
	−110 °C	2408.992	304.272	94.451	1.050
	−170 °C	2738.886	9.966	95.307	0.329
PUF (H)	20 °C	6464.576	443.486	91.093	0.250
	−50 °C	7467.204	328.476	92.208	0.420
	−110 °C	9016.458	895.106	90.669	1.942
	−170 °C	9331.487	603.999	92.927	0.755
PVC-type foam (L)	20 °C	3633.627	36.618	93.106	0.048
	−50 °C	3749.665	66.690	93.889	0.623
	−110 °C	3906.940	426.257	93.335	1.608
	−170 °C	4079.559	66.358	94.803	0.191
PVC-type foam (H)	20 °C	6446.355	464.340	90.407	0.552
	−50 °C	10602.630	374.243	91.952	0.790
	−110 °C	11703.560	426.257	91.283	2.416
	−170 °C	12278.953	559.559	92.893	0.527

## Data Availability

The data presented in this study are available on request from the corresponding author.
